# Manufacturing Process, Microstructure and Physico-Mechanical Properties of W-Cr Coatings Reinforced by Cr3C2 Phase Produced on Tool Steel through Laser Processing

**DOI:** 10.3390/ma16134542

**Published:** 2023-06-23

**Authors:** Dariusz Bartkowski, Aneta Bartkowska

**Affiliations:** 1 Institute of Materials Technology, Faculty of Mechanical Engineering, Poznan University of Technology, ul. Piotrowo 3, 61-138 Poznan, Poland; 2 Institute of Materials Science and Engineering, Faculty of Materials Engineering and Technical Physics, Poznan University of Technology, ul. Jana Pawła II 24, 61-138 Poznan, Poland; aneta.bartkowska@put.poznan.pl

**Keywords:** chromium carbide, laser processing, microstructure, microhardness, corrosion resistance, wear resistance

## Abstract

This paper presents study results of laser processing of W-Cr, WCr/Cr_3_C_2_ and Cr_3_C_2_ pre-coats applied on steel substrate in the form of paste. For this study, production parameters were selected to obtain the greatest possible durability of final coatings. Laser processing was carried out using a diode laser machine with a rated power of 3 kW. The laser beam scanning speed was constant at 3 m/min, but variable laser beam powers were used: 600 W, 900 W and 1200 W. Multiple laser tracks with 60% overlapping were used. After remelting the pre-coat with a steel substrate, new coatings were obtained. Following the experiment, microstructure, microhardness, wear, corrosion resistance and chemical composition were investigated. It was found that it is possible to produce W-Cr/Cr_3_C_2_ coatings through laser processing. These coatings do not have the characteristics of a composite coating; however, increasing the reinforcing phase in the pre-coat positively affects the wear resistance and microhardness. The addition of a reinforcing phase was found to lead to a microhardness of about 750–890 HV01 for 25% and 75% Cr_3_C_2_, respectively, in comparison to coating without Cr_3_C_2_. The wear resistance of coatings reinforced by chromium carbide improved more than twofold in reference to the W-Cr coating.

## 1. Introduction

Surface engineering is a branch of technical sciences dealing with the production and study of modern layers and coatings [1] and mechanical surface treatment, such as shock peening [2] or friction stir processing [3,4]. On the basis of various types of experiments, production methods and techniques can be selected to improve the performance of tools and machine parts. Over the decades, many methods of refining metal surfaces have been developed. Conventional methods of producing layers and coatings, such as carburizing and galvanic methods, are continually being developed. However, with progress in technology modern ways of changing properties of metal surfaces are being developed. These novel methods and techniques mainly lead to the enrichment of surfaces of cheaper and less wear-resistant materials in order to increase their durability. Frequently, surface engineering is also used to increase the hardness and wear resistance of expensive superalloys with very good corrosion resistance but without adequate durability in operation. Typical surface engineering methods include heat treatment (surface hardening) or thermo-chemical treatment, such as diffusion saturation methods (previously mentioned carburization and nitriding, boriding or chromium plating). Among galvanic methods, the most common, such as nickel plating, chromium plating or zinc plating, can be distinguished. The methods that are gaining recognition and being more frequently developed and described by researchers are high-energy techniques, such as laser cladding [5,6,7,8,9,10,11,12,13], plasma arc cladding [14,15] and laser alloying [16,17]. These methods are increasingly used in industry. However, as a large number of their production parameters have multiple influences, they are not used on a large scale. Besides, thorough technological and material knowledge are required. Laser alloyed coatings are mainly used to increase the durability of agricultural tools or those used in petroleum and gas mining. Usually, the purpose of laser cladding or laser alloying is to form a fairly thick coating that is enriched with hard inter-node phases, such as carbides or borides. Laser cladding can also be used as a method of regenerating tools and parts of machines that are exposed to wear.

There are numerous research papers on the production of coatings containing reinforcing phases. They mainly focus on the use of tungsten carbide as a reinforcing phase. There have been attempts to introduce other phases, such as VC, NbC, ZrC and TaC. To that end, various types of generation methods that use high-energy energy sources have been applied. In some attempts, primary phases are introduced directly in the form of carbide particles, while in others pure metal elements and carbon are introduced to produce carbides in situ. In light of the very good diffusion properties of surface layers obtained after diffusion chromium plating processes [18,19,20], it is reasonable to test whether the introduction of a reinforcing phase in the form of chromium carbide will also lead to high hardness and wear resistance. 

Papers on the introduction of a Cr_3_C_2_ phase as a reinforcing phase for the production of coatings are available. In [21], the researchers produced coatings based on 316L steel reinforced with varying Cr_3_C_2_ contents using the laser metal deposition (LMD) method. The test results were compared to the coating without the reinforcing phase. The microhardness of 316L/Cr_3_C_2_ samples were found to double in comparison to 316L coatings. The abrasion resistance of the samples also improved significantly after the addition of Cr_3_C_2_. In [22], the authors produced Cr_3_C_2_-NiCr coatings on H13 steel using laser thermal spraying at three laser beam powers of 1200 W, 1400 W and 1600 W. Moreover, they analyzed the wear resistance at 600 °C and described the wear mechanism. The Cr_3_C_2_-NiCr coatings were shown to consist mainly of Cr_7_C_3_ andCr_3_C_2_ hard phases and NiCr binding phase. The highest laser beam power was found to cause the greatest wear. They were referred to as abrasive and oxidative wear. A similar powder mixture of (Cr_3_C_2_NiCr) was used to produce coatings on a substrate of titanium aluminide (TiAl) [6]. The authors investigated the influences of laser beam power, laser beam scanning speed and powder feed rate on the geometric properties of individual tracks. The microstructure contained various types of chromium carbides (Cr_3_C_2_, Cr_7_C_3_ and Cr_23_C_6_) along with a solid solution of Ni(Cr). The microhardness of the best coating was found to be around 820 HV, which was higher than the substrate of titanium aluminide (320 HV). In [8], the authors present the test results of coatings on an Fe substrate with a high content of V and Cr. These coatings were obtained via laser cladding using a Fe-based powder with varying contents of FeV50. The results showed that the Fe-based coatings were uniform and consisted mainly of a solid solution of α-Fe. With an increase in Cr_3_C_2_ and FeV50, the phases of γ-Fe and V_8_C_7_ were obtained. The microstructure of the coatings demonstrated a typical dendritic structure. An increased concentration of C, V and Cr in the dendritic regions was observed, and the remaining alloying elements were predominantly dissolved in the interdendritic regions. The hardness and wear resistance of Fe-based coatings was increased by the addition of Cr_3_C_2_ and FeV50. The sample with 15% Cr_3_C_2_ and 16% FeV50 showed the highest hardness (1.05 times higher than the sample with 4.5% Cr_3_C_2_ and 5% FeV50). The wear resistance of the former was three times higher. 

Coatings containing Cr_3_C_2_ were also tested for use on parts of motor vehicles, and more specifically on camshafts. A study described in [23] aimed to check the possibility of improving the service life of camshafts. For this purpose, a traditional camshaft was forged from 12CrNi_2_V steel, and then four types of wear-resistant Fe-based powders were designed, introducing different Cr_3_C_2_ and V-rich Fe-based alloy (FeV50) contents into the stainless steel powder. The results showed that gradient materials formed a good metallurgical connection. The composition of the phases consisted mainly of α-Fe, Cr_23_C_6_ and V_2_C phases. With the addition of Cr_3_C_2_ and FeV50, the average microhardness and abrasion resistance of the gradient materials were significantly better than 12CrNi_2_V. In [24], hard Fe-Cr_3_C_2_ coatings with varying Cr_3_C_2_ contents were produced on a 35CrMo steel substrate through laser cladding. The results of the experiments showed that the coatings were uniform, continuous and crack free. The coating microstructures consisted mainly of austenitic dendrites and M_7_C_3_. The microhardness gradually increased from the bottom to the top of the coating and increased with the increase in Cr_3_C_2_ content. Fe-Cr_3_C_2_ coatings showed improved wear resistance compared with the substrate. The authors defined the mechanism of wear as spalling fatigue. The substrate on which coatings containing Cr_3_C_2_ were produced was also titanium alloy [12]. The authors set the goal of increasing the surface hardness in light of the low hardness common for this type of alloy. Therefore, Ti(C,B)/Ni coatings with varying Cr_3_C_2_ contents on the surface of Ti-6Al-4V were prepared. The average microhardness of the cladding layer without the addition of Cr_3_C_2_ was found to be 896.7 HV0.2, which is approximately 1.5 times higher than that of the Ti-6Al-4V substrate (360 HV0.2). With an increase in Cr_3_C_2_ content (10, 20, 30% by weight), the average microhardness of the coating increased to 948.7, 990.4 and 1035.8 HV0.2, respectively. Therefore, the average hardness of the coating with the addition of 30% by weight of Cr_3_C_2_ is 15.5% was proved to be higher than that of the coating without Cr_3_C_2_. A simultaneous increase in wear resistance was also observed. In [12], an attempt was made to increase the wear resistance of the steel substrate by producing a VC-Cr_7_C_3_ coating in situ using a pre-applied powder consisting of vanadium, carbon and high-carbon ferrochrome. The authors obtained an average coating hardness of around 1050 HV. Wear tests showed that the wear resistance of the coating produced was 4 times higher than that of a steel substrate. In addition to laser processing, plasma processes were studied. In [25], plasma spraying with carbides was studied. Two types of carbides, WC and Cr_3_C_2_ and their mixture in the 50:50 weight ratio, were used. The coatings were subjected to erosion tests in an aluminum oxide stream and in a 3.5% *w*/*w* sodium chloride (NaCl) solution. The coating made of a carbide mixture was found to have the highest hardness, reaching 1350.2 HV. These coatings also showed higher resistance to erosive wear. However, the authors emphasized that the Cr_3_C_2_ coatings were characterized by better corrosion resistance due to their passivation. 

An analysis of the literature leads to the conclusion that the topic of the production of coatings containing Cr_3_C_2_ is of interest to researchers. There have been attempts to produce in situ coatings and to introduce a reinforcing phase as a powder particle. Coatings were produced on multiple substrates, including steel. Laser cladding or spraying and in situ methods were used. However, it is justifiable to test the properties of coatings produced by remelting the pre-coat in the form of a paste. As very little information on this manufacturing method is available, the tests were carried out on Cr_3_C_2_-reinforced coatings produced through laser remelting of the pre-coat. In order to simultaneously increase the mechanical properties and ensure corrosion resistance, the authors’ original W-Cr matrix was used. This paper describes tests involving laser remelting pre-coats W-Cr, W-Cr/Cr_3_C_2_ and Cr_3_C_2_ and the testing of their properties. Further, tests on microstructure, microhardness, wear, corrosion resistance and chemical composition were carried out.

## 2. Materials and Methods

W-Cr/Cr_3_C_2_ coatings were produced using laser processing of pre-coat in the form of paste applied on 145Cr6 steel. The pre-coat consisted of three types of powders: tungsten, chromium and chromium carbide. The chemical composition of 145Cr6 steel is given in Table 1. Rectangular specimens with dimensions of 20 mm × 20 mm × 6 mm were used in this study.

**Figure 1:**
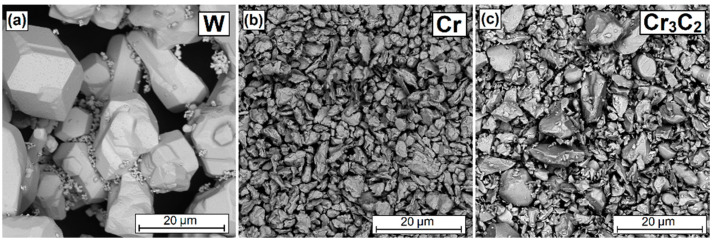
Morphology of the powder particles used: W (**a**), Cr (**b**) and Cr_3_C_2_ (**c**).

The shape and size of the individual components of the powder mixture necessary to prepare the pre-coat are presented in Figure 1. An average particle size (APS) was in each case < 12 µm, which was in accordance with the delivered certificates. The tungsten powder purity was 99.9%, and the chromium powder purity was 99.2%. All the presented parameters are in accordance with the producer’s data (Sigma-Aldrich, St. Louis, MO, USA). 

**Figure 2:**
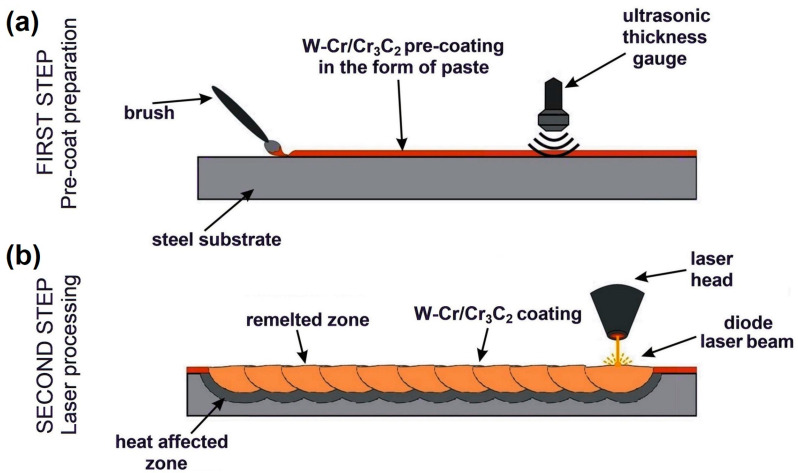
Scheme of W-Cr/Cr_3_C_2_ coating production using laser processing: (**a**) pre-coat application and ultrasonic testing of thickness, (**b**) laser processing and carbide coating obtained.

The procedure to produce the W-Cr/Cr_3_C_2_ coating is shown in Figure 2. The process was divided into two stages. In the first stage, a pre-coat in the form of a paste was applied on the surface of the steel as delivered (without heat treatment). Powder mixtures were prepared prior to paste preparation. The base of each pre-coat was a mixture of tungsten and chromium powders. Cr_3_C_2_ was then added to this mixture in weight ratios of 25%, 50% and 75%, respectively. Additionally, pre-coats were also made from only W-Cr and from only chromium carbide Cr_3_C_2_. The paste was made by adding an aqueous solution of sodium Na_2_O + SiO_2_ silicic acid and distilled water to the powder mixtures (JURGA, Zbrudzewo, Poland). The amounts of individual components were selected so that for 10 g of powder mixture there were 2 mL of Na_2_O + SiO_2_ and 2 mL of distilled water. The paste was stirred mechanically until a homogeneous consistency was obtained, allowing the paste to be applied to steel surfaces with a brush. To facilitate the breaking of the resulting agglomerates, the container in which the mixing was performed was placed in an ultrasonic washer. For all the samples, pre-coats with a thickness of 150 μm were made, and the thickness of each sample was checked with an ultrasonic coating thickness meter (Elcometer, Manchester, UK). This measurement was made after the pre-coat had dried, and samples on which a thicker or thinner coating was produced were discarded and not submitted for laser processing. Such an approach was adopted due to the imperfect method of applying the paste with a brush, which was manifested by difficulties in monitoring coating thicknesses. 

In the second stage, the selected samples with pre-coatings applied were subjected to laser processing. For this purpose, a continuous diode laser beam generated by the TruDiode 3006 with a nominal power of 3 kW (TRUMPF, Ditzingen, Germany) was used. The laser beam’s movement was controlled through the integrated KR16-2 robotic arm (KUKA, Augsburg, Germany). The robotic arm enabled changes in the laser beam’s position and speed. A schematic of the manufacturing process of the W-Cr/Cr_3_C_2_ coating divided into two stages is shown in Figure 2. In laser processing, a constant laser beam speed of 3 m/min was used. To determine the influence of the laser beam’s power on the properties of the coatings obtained, three powers were used: 600 W, 900 W and 1200 W. The diameter of the laser beam diameter used in the study was 1 mm, while its wavelength was 1040 nm. The laser beam was characterized by the transverse electromagnetic mode TEM00. Carbide coatings were made in such a way that the entire sample surface was laser processed. Therefore, a 60% laser track overlap was used, in accordance with the formula used in [26]. During laser processing, the laser beam was moved from one edge of the steel specimen to the opposite edge and then was turned off. Afterwards, the laser head was returned to the start point. In the next step, the laser head was transferred perpendicular to the previous move at a distance of 0.4 mm, and then the laser beam move was repeated. This was repeated until the coating was produced on the entire surface of the specimens. As mentioned earlier, a total of five types of pre-coats were used in the tests, which at three different laser beam powers yielded 15 different carbide coatings of varying properties (Table 2). 

Microstructure observations of the coatings were carried out on etched cross-sections using both the Huvitz HRM-300 light microscope (Huvitz Corp., Burim-ro, Republic of Korea) and the MIRA-3 scanning electron microscope (TESCAN, Brno, Czech Republic). The scanning electron microscope is equipped with an EDS-UltimMax X-ray dispersion spectrometer (Oxford Instruments, High Wycombe, UK) and dedicated Aztec Energy Live Standard software. Prior to the observation of the microstructure, all the samples were ground and polished using the Mecatech 250 device (PRESI, Eybens, France). The abrasive discs recommended by the manufacturer of the device (marked as materials for grinding and polishing hard steels) were used for this purpose. The procedure of final polishing of the cross-sectional surfaces consisted of four steps. The following grain sizes were used: step 1: 54 µm; step 2: 18 µm; step 3: 3 µm; and step 4: 0.8 µm. All cross-sections were etched in 5% HNO_3_ solution for several seconds. Analysis of the chemical composition of W-Cr/Cr_3_C_2_ coatings was carried out with EDS, using the point analysis method. Microhardness tests of the W-Cr/Cr_3_C_2_ coatings were carried out on cross-sections from the coating surface to the steel substrate along a straight line passing through the central area of the laser track. 

Microhardness tests were performed using the Vickers method with the FM-810 microhardness tester (Future-Tech, Kawasaki, Japan) equipped with the FT-Zero automatic dent measurement software. The tests were carried out under a load of 100 g. The loading time was 15 s. Corrosion resistance tests were carried out using the ATLAS 1131 EU&IA device (Atlas-Sollich, Rębiechowo, Poland) in a 3.5% NaCl aqueous solution, as recommended in the PN-EN ISO 17475:2010 standard [27]—Corrosion of metals and alloys—Electrochemical test methods—Guidelines for the performance of potentiostatic and potentiodynamic polarization measurements (translation from Polish). The potentiodynamic method was used, through which the anode polarization curves were obtained. Corrosion resistance tests were carried out at 22 °C with a scanning rate of 0.5 mV/s. A saturated calomel electrode was used as the reference electrode, and a platinum electrode as an auxiliary electrode. On the basis of corrosion resistance tests, the corrosion potential and corrosion current of the analyzed coatings were determined. Wear resistance tests were carried out on 6 mm × 6 mm × 6 mm square samples cut from the central part of previously produced samples with W-Cr/Cr_3_C_2_ coatings using a wire EDM machine. This area was selected in order to maximize laser track repeatability in this area. The Amsler method was used to conduct wear resistance tests. The tests were performed using the MBT-01 tribotester (MBT, Poznań, Poland). Ring-shaped counter samples were made of C45 steel after quenching in water at 850 °C and tempering at 180 °C for 1 h. The outer diameter of the counter-sample was 20 mm, and the inner diameter was 12 mm. The width of the ring was 12 mm. These tests were carried out under dry friction conditions with the following parameters: counter-sample speed: 250 rpm; load: 98 N; friction time: 60 min. Weight loss of the samples was measured on the analytical balance AS220.R2 (RADWAG, Radom, Poland) following every 10 min of wear test. After wear tests, observations of wear surface condition were carried out using scanning microscopy.

## 3. Results and Discussion

### 3.1. Microstructure and Chemical Composition

**Figure 3:**
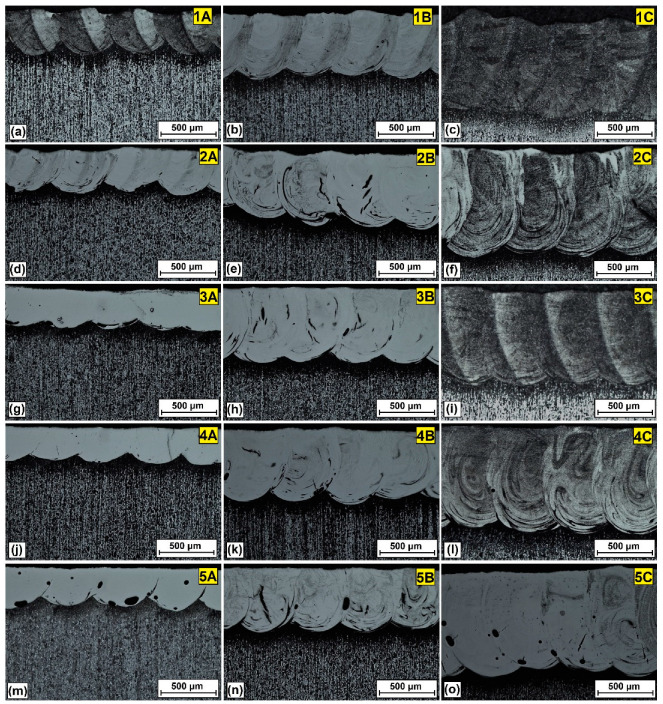
Morphology of the produced coatings: W-Cr (**a**–**c**), W-Cr/Cr_3_C_2_ (**d**–**l**) and Cr_3_C_2_ (**m**–**o**). Designations of the parameters used in accordance with Table 2.

As a result of laser beam operation, the pre-coats containing tungsten, chromium and chromium carbide or any of these components individually were mixed with an iron-based alloy substrate. This resulted in the formation of new coatings that were not previously produced by other researchers. Figure 3 shows the morphology of the W-Cr/Cr_3_C_2_ coatings obtained. For easier identification of individual production parameters, Figure 3a–o is marked in the same way as in Table 2. The chemical compositions of the pre-coatings (1—100% W-Cr, 2—W-Cr/25% Cr_3_C_2_, 3—W-Cr/50% Cr_3_C_2_, 4—W-Cr/75% Cr_3_C_2_, 5—100% Cr_3_C_2_) were marked with numbers, whereas laser the beam powers used (A—600 W, B—900 W, C—1200 W) were marked with letters. The effect of increasing laser beam power on the average thickness of the obtained coatings is clearly visible. In each case, a twofold increase in laser beam power resulted in an at least twofold increase in coating thickness, as in the case of the 100% W-Cr coating (Table 3). The addition of Cr_3_C_2_ carbide particles to the W-Cr matrix enhanced the effect of increasing the coating thickness. In the coating containing 75% Cr_3_C_2_, its average thickness was almost three times greater, at 1200 W of laser beam power compared to 600 W. It can therefore be stated unequivocally that the addition of Cr_3_C_2_ particles to the W-Cr matrix contributes to the production of thinner coatings. However, it should be noted that the values given in Table 3 are averages, calculated from 10 measurements made along the laser tracks axis, and the dimensions of these tracks increase as the substrate heats up due to the heat generated by the laser beam. In order to produce coatings in which each laser track is characterized by a uniform thickness, it is necessary to monitor the sample temperatures in real time and adjust the laser beam power and/or its speed accordingly. Laser track sizes are also affected by the thickness of pre-coatings. In light of the method of applying pre-coat, special attention should be paid to whether its thickness is the same over the entire sample surface. Pre-coatings applied with a brush are characterized by unevenness resulting from the very procedure of their production. Increasing the amount of chromium carbide particles was found to result in an increasingly smaller coating thickness when using the lowest laser beam power. This is because heat was absorbed by the carbides, which are characterized by a greater heat capacity. Such a relationship did not appear with higher laser beam powers. This is most likely because the carbides were completely remelted in a shorter time and could not receive any more heat. For a coating containing 100% Cr_3_C_2_, there was a sudden increase in coating thickness obtained at low power. However, it should be noted that these coatings are porous, which may have affected the formation of individual tracks.

**Figure 4:**
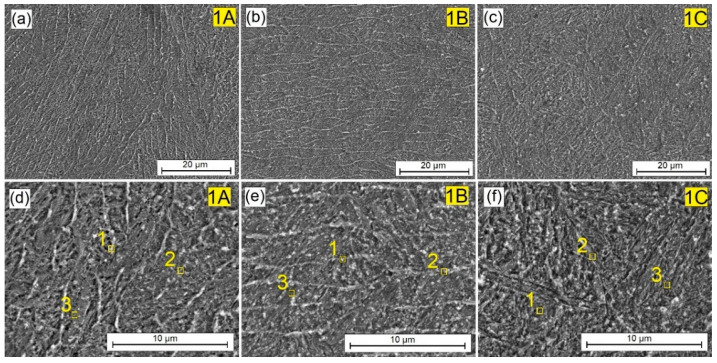
Microstructure of 100% W-Cr coatings produced using laser processing of pre-coat and the following beam powers: (**a**) 600 W, (**b**) 900 W, (**c**) 1200 W, (**d**) 600 W higher magnification, (**e**) 900 W higher magnification, (**f**) 1200 W higher magnification.

Figure 4 shows the central areas of the remelted zone of laser tracks obtained by laser alloying the pre-coat made of exclusively matrix elements, i.e., tungsten and chromium. In the coating produced at a laser beam power of 600 W and 900 W the presence of a bright mesh of an elongated shape was found, which became the focus of an increased concentration of elements added to the steel substrate, as confirmed by the EDS test results presented later in the article. Each pre-coat produced remelted with the steel substrate. This resulted in the formation of a compact surface layer well combined with the steel substrate. Increasing the laser beam power to 1200 W resulted in intensive mixing of the pre-coat with the iron from the substrate and thus the disappearance of the previously visible bright mesh. The microstructure obtained was needle-like (martensite) and homogeneous throughout the studied area. This was associated with an increase in iron content in the produced coating.

**Figure 5:**
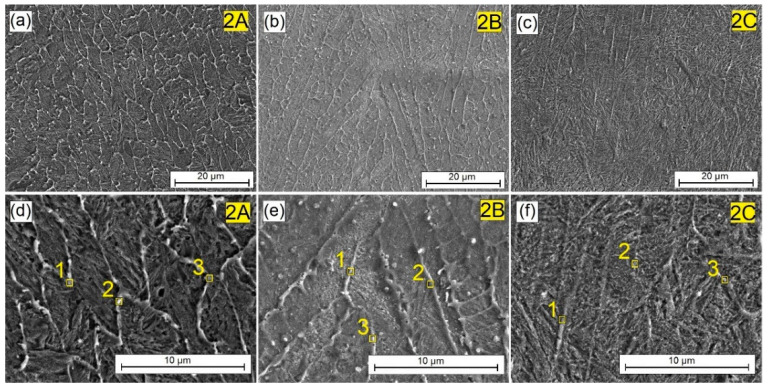
Microstructure of W-Cr/25% Cr_3_C_2_ coatings produced using laser processing of pre-coat and the following beam powers: (**a**) 600 W, (**b**) 900 W, (**c**) 1200 W, (**d**) 600 W higher magnification, (**e**) 900 W higher magnification, (**f**) 1200 W higher magnification.

**Figure 6:**
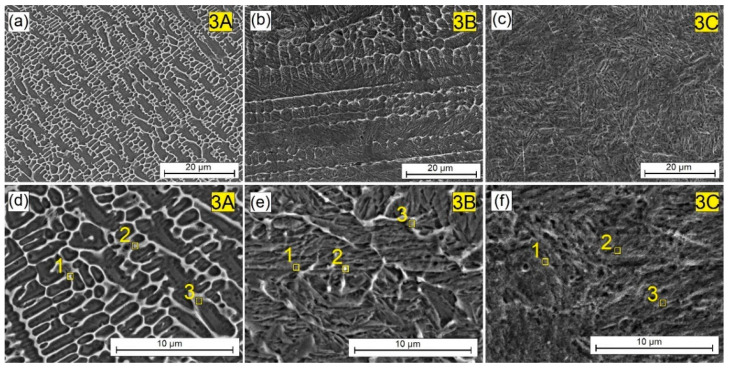
Microstructure of W-Cr/50% Cr_3_C_2_ coatings produced using laser processing of pre-coat and the following beam powers: (**a**) 600 W, (**b**) 900 W, (**c**) 1200 W, (**d**) 600 W higher magnification, (**e**) 900 W higher magnification, (**f**) 1200 W higher magnification.

**Figure 7:**
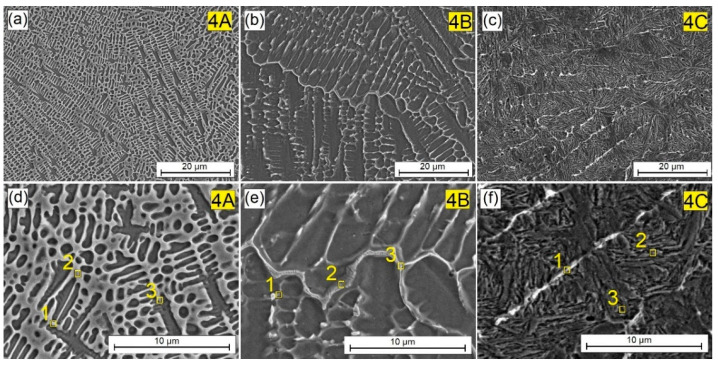
Microstructure of W-Cr/75% Cr_3_C_2_ coatings produced using laser processing of pre-coat and the following beam powers: (**a**) 600 W, (**b**) 900 W, (**c**) 1200 W, (**d**) 600 W higher magnification, (**e**) 900 W higher magnification, (**f**) 900 W higher magnification.

Figure 5, Figure 6 and Figure 7 show the surface layers produced by remelting with the steel substrate of the pre-coats varying amounts of Cr_3_C_2_ chromium carbide particles: 25% (Figure 5), 50% (Figure 6), and 75% (Figure 7), respectively. The significant influence of the amount of reinforcing phase added to the microstructure of the surface layer produced was observed. On the other hand, structural changes had a key influence on the mechanical, physicochemical and operational properties obtained. In the surface layer produced at 25% Cr_3_C_2_ in the W-Cr matrix, a structure similar to that obtained without the reinforcing phase was found. When the lowest laser beam power (600 W) was used, the resulting mesh (Figure 5a,d), however, was more clearly visible, and its shape was not as elongated. The mesh was arranged in a cell-like shape. Increasing the laser beam power to 900 W caused the mesh to lengthen and its cell-like character to disappear. Increasing the laser beam power increased the proportion of iron in the surface layer and simultaneously reduced the influence of Cr_3_C_2_ particles on the microstructure. The use of a maximum laser beam power of 1200 W resulted in a microstructure similar to that obtained using the same power and the W-Cr pre-coat but without the reinforcing phase. Therefore, in order to obtain significant structural changes, parameters that will not cause a significant remelting of the steel substrate should be used. 

As for the surface layer obtained by remelting the pre-coat W-Cr/50% Cr_3_C_2_, the changes in the microstructure were more pronounced. At 600 W of laser beam power, a very extensive cellular-dendritic structure with a light coloration against the background of a dark matrix was observed. Increasing the laser beam power initially dispersed the light-colored areas while creating a needle-like structure in the matrix. The increase in laser beam power to the maximum values caused the microstructure to change to a needle-like format without visible bright areas in the form of mesh or dendrites. Therefore, this confirmed that the laser beam power, and thus the heat delivered, has a key influence on the microstructure obtained. 

In order to check whether the increase in the amount of the reinforcing phase in the W-Cr matrix will result in a similar effect, but on a smaller scale, tests were carried out on samples with the pre-coat W-Cr/75% that Cr_3_C_2_ produced. The microstructures obtained are shown in Figure 7. At the lowest laser beam power, a cellular dendritic structure was obtained with a much greater fragmentation than in the layer containing 50% Cr_3_C_2_ (Figure 7a,d). There is a significant share of light-colored structural elements. Increasing the laser beam power to 900 W (Figure 7b,e) resulted in a reduction in the intensity of the cellular dendritic structure, as in the case of the previously described coatings containing a smaller amount of the reinforcing phase. The use of the maximum laser beam power of 1200 W (Figure 7c,f) resulted in greater mixing of the pre-coat with the steel substrate, which resulted in the presence of a needle-like structure. However, residual remains of light-colored mesh were observed. Therefore, it can be concluded that the increase in the share of the reinforcing phase in the pre-coating increases the number of light-colored precipitates, regardless of the laser beam power used. However, it should be remembered that there is a certain borderline limit on the amount of the reinforcing phase that will leave a light-colored phase when a high laser beam power is used.

**Figure 8:**
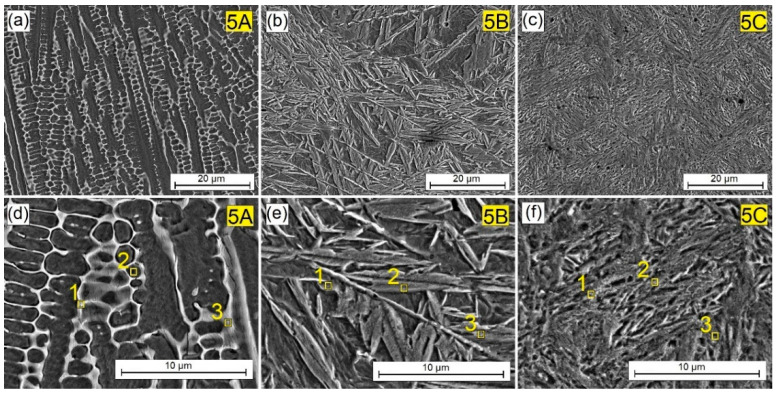
Microstructure of 100% Cr_3_C_2_ coatings produced using laser processing of pre-coat and the following beam powers: (**a**) 600 W, (**b**) 900 W, (**c**) 1200 W, (**d**) 600 W higher magnification, (**e**) 900 W higher magnification, (**f**) 1200 W higher magnification.

While observing structural changes when the amount of the reinforcing phase in the W-Cr matrix increased, the effect the matrix itself had on the construction of the new structure was also observed. For this purpose, samples without the W-Cr matrix were prepared and compared with previous results. The pre-coat consisted entirely of particles of the Cr_3_C_2_ reinforcing phase. The resulting microstructure is shown in Figure 8. The W-Cr matrix was found to have a significant influence on building a cellular dendritic structure. The absence of tungsten and chromium resulted in a significant reduction in the proportion of the W-CR matrix, or even its complete elimination at higher laser beam powers. The increase in power to 900 W resulted in the complete replacement of the bright precipitates with martensitic needle-like structures. Increasing the power to 1200 W caused fragmentation of the needle-like structure. This is because chromium carbide was introduced into the steel surface. 

Under the influence of the laser beam, the steel surface was enriched with carbon and chromium. The reinforcing phase particles were too small and became completely remelted. Both carbon and chromium increased the steel hardenability, which results in a needle-like structure produced by hardening. When using 1200 W of laser beam power, the degree of supercooling was so large that the structure became fine-grained.

By analyzing the images of the microstructures presented in Figure 4, Figure 5, Figure 6, Figure 7 and Figure 8 and bearing in mind the input parameters, including the size of the reinforcing phase particles and the methodology of the coating production, it can be concluded that the use of small particles of the reinforcing phase will not lead to the production of a composite structure. It is true that a new structure will be obtained, in which the dendritic precipitates and matrix can be identified. However, such a structure cannot be treated as a composite structure, where the phases are clearly separated from each other, as was obtained in previous studies on other types of carbides [10,11,17]. However, it can be concluded that the use of both the W-Cr matrix and the Cr_3_C_2_ reinforcing phase has a significant impact on building a new structure, and that the careful selection of the laser beam parameters can control the shape of the individual structural elements. Structural changes also affect the physicochemical properties of the coatings. This is why the chemical composition study using point EDS was carried out. The results of the chemical composition tests for individual coatings are presented in Table 4, Table 5, Table 6, Table 7 and Table 8. 

The measurement points are indicated by the numbers in Figure 4, Figure 5, Figure 6, Figure 7 and Figure 8, showing the enlargement of the remelted area. Table 4 presents the chemical composition of the W-Cr coating, without the reinforcing phase, produced at three laser beam powers. There was an increased presence of tungsten and chromium in the light-colored areas. The dark areas no longer contain such amounts of these elements. However, a high carbon content results from the EDS methodology used. These values should be treated with caution. Increasing the laser beam power was found to cause the content of chromium and tungsten to decrease significantly. In both cases, these values are almost halved at 900 W of beam power, and about five times lower at 1200 W of beam power. Because the pre-coat containing carbide-forming materials was remelted with the substrate, the phases of the complex carbides were most likely formed. The carbon necessary for their formation could have come from the steel substrate. Table 5 shows the chemical composition of the W-Cr coating reinforced with 25% Cr_3_C_3_ particles by weight. The results are presented for the three laser beam powers. As before, there was an increased presence of tungsten and chromium in the light-colored areas. However, these values were lower. This could be due to the lower amount of tungsten and chromium in the pre-coating. These elements were partially replaced with chromium carbide particles. However, the correlations observed earlier were unchanged. As the power of the laser beam increased, the amount of chromium and tungsten decreased.

Table 6 shows the chemical composition of the W-Cr coating reinforced with 50% Cr_3_C_3_ particles by weight. An increased content of tungsten and chromium was found, reaching a value of 19.14 wt.% Cr and about 9 wt.% W. These values were recorded for bright areas on the microstructure shown in Figure 6d. As the laser beam power increased, the content of chromium and tungsten decreased with a simultaneous increase in iron and carbon. This may indicate the formation of complex carbide phases containing iron. Increasing the laser beam power to 1200 W led to a more than tenfold reduction in chromium content. The tungsten content decreased by approximately four times. 

Table 7 shows chemical composition of the W-Cr coating reinforced with 75 wt.% Cr_3_C_2_ particles. The conclusions were very similar to those in which 50% Cr_3_C_2_ was used; however, the chromium and tungsten contents were higher and reached values of 24 wt.% and approximately 6.5 wt.%, respectively. The study areas are marked with yellow squares in Figure 7d–f. As the laser beam power increased, the chromium and tungsten contents decreased, and their lowest values were approximately 2 wt.% Cr and 1.5 wt.% W. 

Table 8 shows the chemical composition of the coating produced by remelting a pre-coat containing 100% Cr_3_C_2_ particles. The EDS test of chemical composition showed residual amounts of tungsten, which should be treated as an instrument error as tungsten was not added in the laser remelting process. Moreover, the substrate should not contain tungsten. However, up to about 18 wt.% of chromium was found. Because no additional matrix was provided, the chromium carbides were remelted directly with the steel substrate. Increasing the laser beam power reduced the presence of chromium in the tested area. The areas that were analyzed are marked with yellow squares in Figure 8.

### 3.2. Microhardness

**Figure 9:**
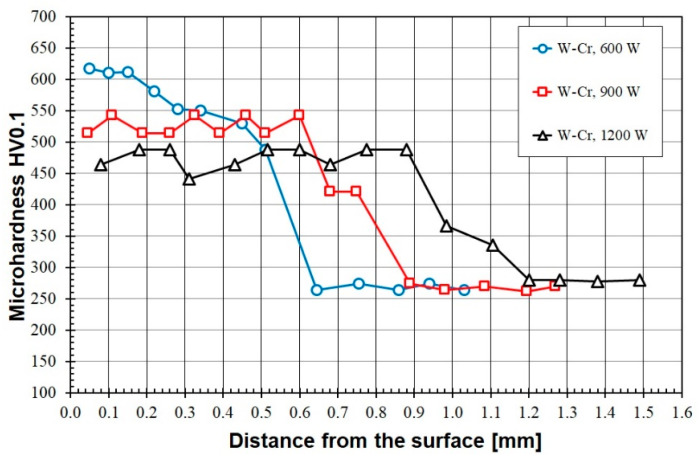
Microhardness of the W-Cr coatings produced using laser beam powers of 600 W, 900 W and 1200 W.

**Figure 10:**
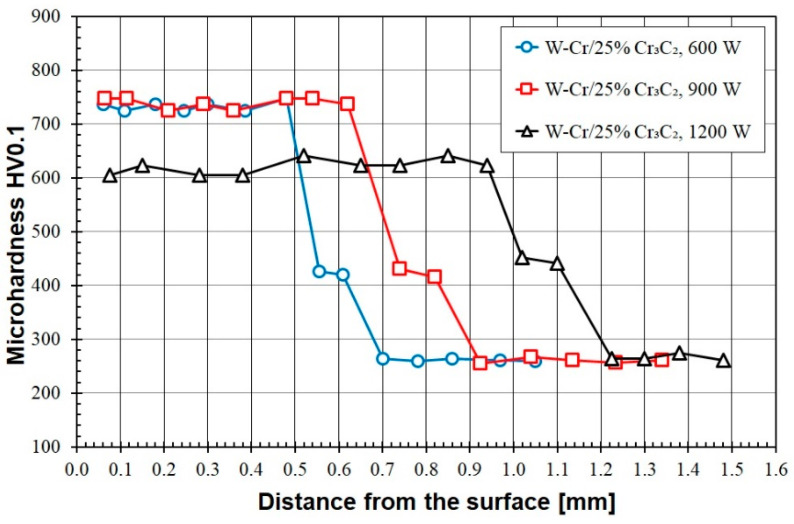
Microhardness of the W-Cr/25% Cr_3_C_2_ coatings produced using laser beam powers of 600 W, 900 W and 1200 W.

**Figure 11:**
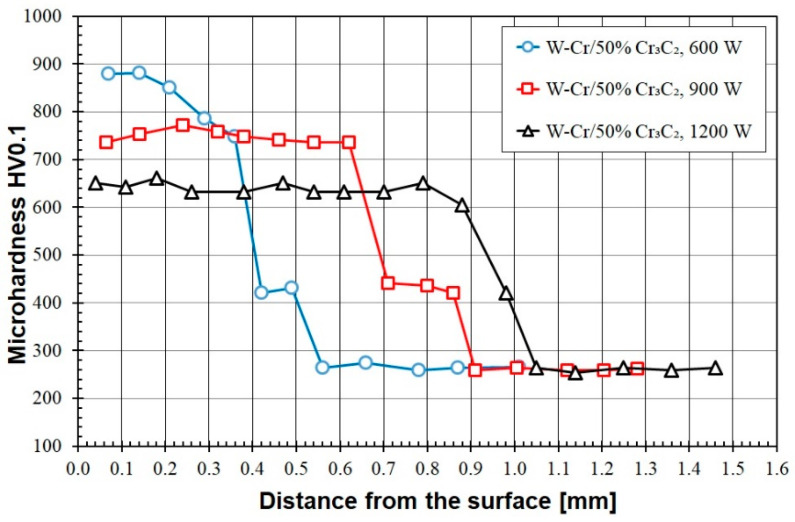
Microhardness of the W-Cr/50% Cr_3_C_2_ coatings produced using laser beam powers of 600 W, 900 W and 1200 W.

**Figure 12:**
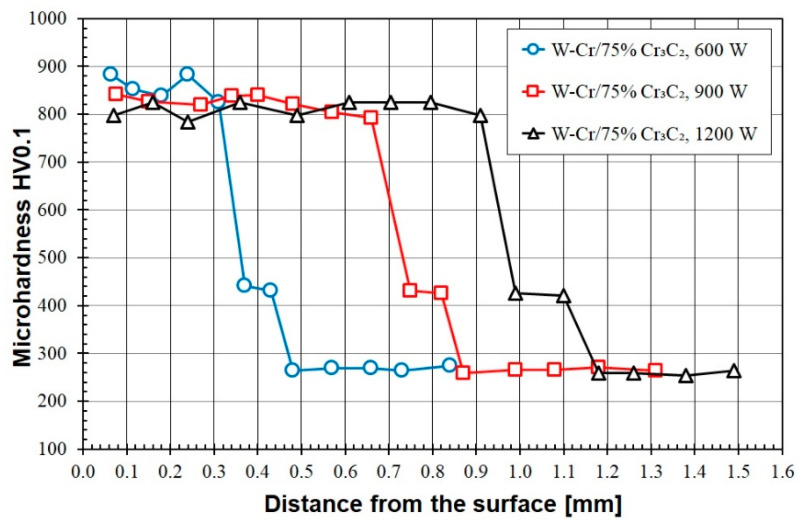
Microhardness of the W-Cr/75% Cr_3_C_2_ coatings produced using laser beam powers of 600 W, 900 W and 1200 W.

**Figure 13:**
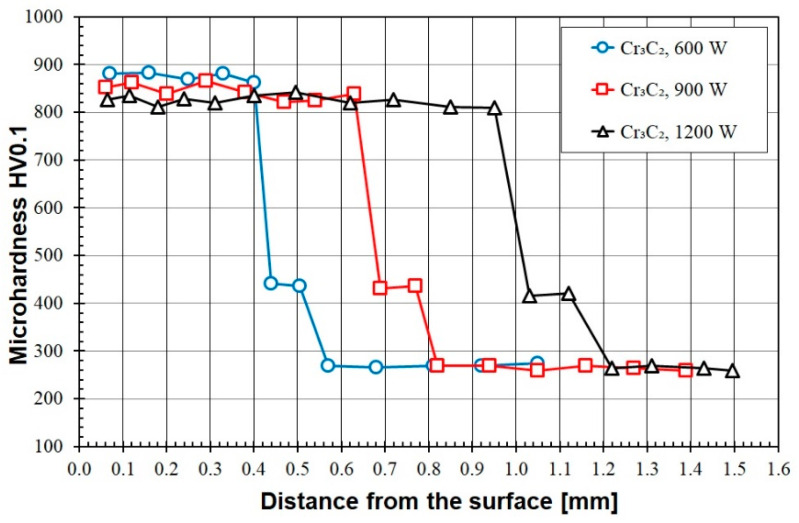
Microhardness of the Cr_3_C_2_ coatings produced using laser beam powers of 600 W, 900 W and 1200 W.

Figure 9, Figure 10, Figure 11, Figure 12 and Figure 13 show the results of microhardness measurements and present them as hardness profiles from the sample surface to the substrate. There are relationships between the obtained microhardness values, the chemical composition of the pre-coat and the laser beam power. Figure 9 shows the results of the microhardness measurements of W-Cr coatings without the addition of a reinforcing phase. The maximum hardness obtained is approximately 620 HV0.1. This hardness was obtained with a laser beam power of 600 W and was maintained at a depth of about 150 µm. Then the hardness decreased to 480 HV in the heat-affected zone. Increasing the laser beam power to 900 W resulted in a decrease in hardness, and the measurements were in the range of 500–550 HV0.1. However, an advantage of increasing the laser beam power was that the thickness of the coating, including the heat-affected zone, was increased. The use of 1200 W of laser beam power led to a decrease in hardness; the results obtained were less than 490 HV0.1. Therefore, it can be concluded that the increase in laser beam power negatively affects the microhardness values. 

Enrichment of the W-Cr pre-coat with a reinforcing phase in the form of Cr_3_C_2_ particles led to an increase in microhardness. Figure 10 shows the microhardness profile obtained for the W-Cr/25% Cr_3_C_2_ coating. With a laser beam power of 600 W, a microhardness of approximately 740 HV0.1 was obtained. One disadvantage observed was a sudden decrease in hardness in the heat-affected zone. Interestingly, increasing the laser beam power to 900 W did not reduce the microhardness in any way but contributed to an increase in the thickness of the coating. However, the reduction in hardness resulted in an increase in laser beam power to 1200 W. The hardness of this coating oscillated around 600 HV0.1. 

An increase in the content of the Cr_3_C_2_ reinforcing phase did not affect the microhardness of the coating produced at a laser beam power of 900 W (Figure 11). In this case, the hardness still oscillated around 750 HV0.1. However, increasing the amount of chromium carbide influenced the remaining coatings. The hardness of the coating produced with the laser beam power of 600 W increased significantly as the values of approximately 890 HV0.1 were achieved there. This hardness gradually decreased towards the substrate. The microhardness of the W-Cr/50% Cr_3_C_2_ coating produced with a laser beam power of 1200 W also slightly increased and reached values of approximately 650 HV0.1. 

Figure 12 shows the microhardness measurement results of coatings produced at three different laser beam powers and with a pre-coat of W-Cr/75% Cr_3_C_2_. In this case, the increase in the amount of the reinforcing phase did not significantly affect the hardness obtained with the laser beam power of 600 W. However, the hardness increased significantly with the laser beam powers of 900 W and 1200 W. Compared to the value of 50% Cr_3_C_2_, the hardness increased by about 100 HV0.01 for 900 W and by about 150 HV0.1 for 1200 W. It can therefore be assumed that the pre-coat containing 25% W-Cr and 75% Cr_3_C_2_ is the most favourable in terms of obtaining high hardness. At the same time, it must be noted that in this pre-coat a uniform hardness is obtained over almost the entire thickness of the coating produced. In the case of products where their size does not matter but their service life does, these are the best coatings among the W-Cr/Cr_3_C_2_ coatings tested. Typical applications may be in mining and agriculture, where a tool (e.g., used in soil) does not have to meet stringent dimensional criteria but is intended to remain usable for as long as possible.

The microhardness test was also performed on coatings produced by remelting the pre-coat containing only Cr_3_C_2_ (Figure 13). In this case, the results were very similar to those obtained for the W-Cr/75% Cr_3_C_2_ coatings. The microhardness was in the range of 800–900 HV0.1, and increasing the laser beam power reduced the microhardness values.

### 3.3. Wear Resistance

**Figure 14:**
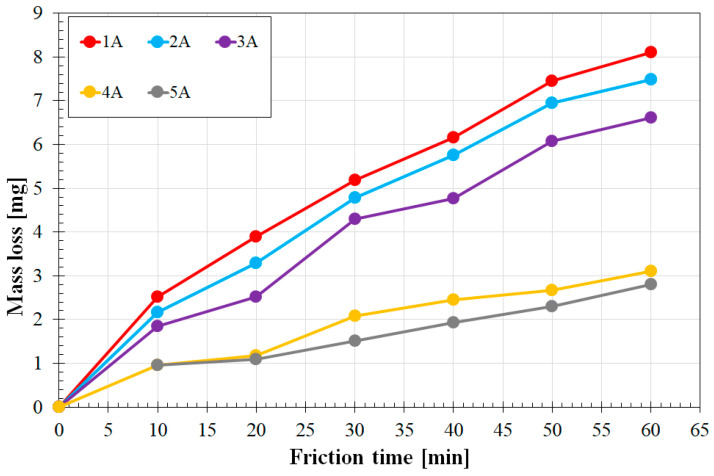
Wear resistance coatings produced using a laser beam power of 600 W.

**Figure 15:**
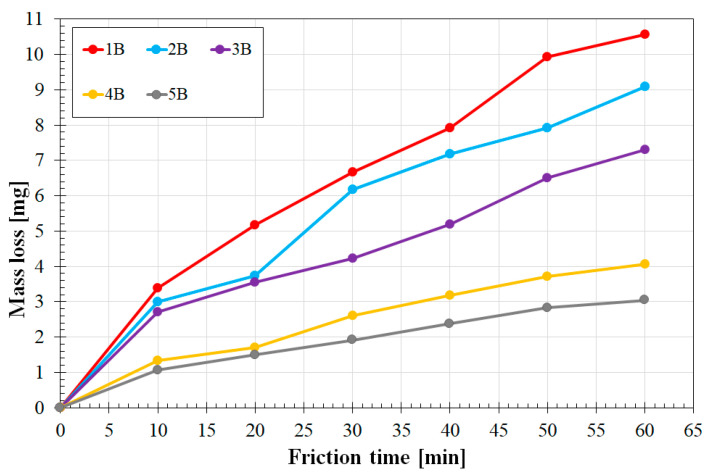
Wear resistance coatings produced using a laser beam power of 900 W.

**Figure 16:**
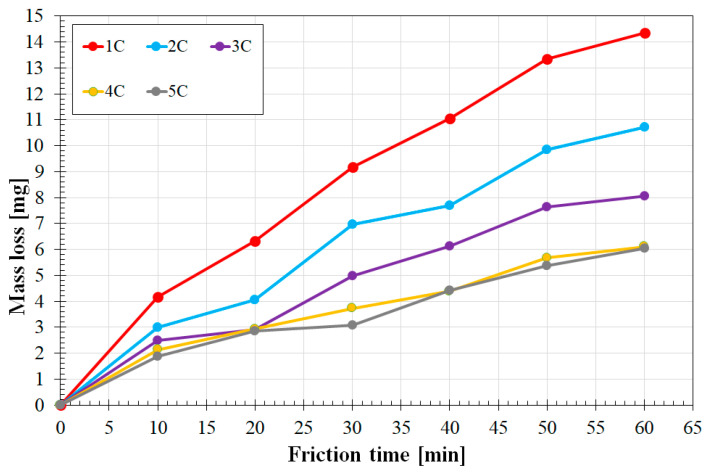
Wear resistance coatings produced using a laser beam power of 1200 W.

The surface layers formed by remelting the pre-coatings W-Cr, W-Cr/Cr_3_C_2_ and Cr_3_C_2_ were subjected to a wear resistance test under dry friction conditions. The results in the form of graphs illustrating the correlation between weight loss and friction time are presented in Figure 14, Figure 15 and Figure 16 for the laser beam powers of 600, 900 and 1200 W, respectively. Friction wear resistance was found to be correlated with the microhardness obtained. Figure 14 shows the results of wear resistance tests for all the coatings produced using the lowest laser beam power of 600 W. The coatings produced by remelting the pure reinforcing phase Cr_3_C_2_ and the coatings containing 75% of this phase were found to have the highest wear resistance. In earlier studies, researchers often found a deterioration in wear resistance due to an increase in the amount of the reinforcing phase. However, this was due to the composite nature of the coatings. The phases were then chipped from the coatings, which also caused microcutting of the matrix. In the present case, the Cr_3_C_2_ particles were small enough to completely remelt, which did not bring about the effect of chipping particles from the matrix, and the secondary precipitates of carbide phases significantly improved the wear resistance. Reducing the amount of the phase to 50% significantly deteriorated the wear resistance. This was the result of a lower amount of carbide phases in the matrix. In the W-Cr coatings without a reinforcing phase, the wear resistance was the lowest, which was the effect of a negligible number of carbide phases. A wear resistance almost three times higher was observed for the coatings produced by remelting a pre-coat with a high amount of Cr_3_C_2_ compared to the W-Cr coatings.

Figure 15 shows the results of wear resistance tests for all the variants of the coatings produced with a laser beam power of 900 W. A similar trend was observed for the coatings produced with a power of 600 W. However, an increase in laser beam power resulted in an overall decrease in wear resistance. As for coatings containing the highest amount of reinforcing phase (75% Cr_3_C_2_), the weight loss increased by about 1 mg compared to the coating produced at 600 W. In a coating not containing the reinforcing phase, the weight loss increased by about 2.5 mg. Therefore, it can be concluded that an increase in laser beam power by 300 W results in an approximately 30% reduction in wear resistance compared to coatings produced at 600 W. 

Figure 16 shows the results of wear resistance tests of the W-Cr, Cr_3_C_2_ and Cr_3_C_2_ phase-reinforced W-Cr coatings produced with the highest laser beam power of 1200 W. A very similar tendency was observed in achieving wear resistance related to the composition of the reinforcing phase. The greater the reinforcing phase, the greater the wear resistance. However, there was no sudden change in wear resistance when comparing the coatings containing 75% Cr_3_C_2_ and 50% Cr_3_C_2_, as could be observed among coatings produced with 600 W of laser beam power. Almost the same wear resistance was found among the coatings W-Cr/75% Cr_3_C_2_ and 100% Cr_3_C_2_. An increase in the laser beam power by another 300 W was found to increase the weight loss by 3.5 mg (for the W-Cr coating) and by 2 mg (for the W-Cr coating/75% Cr_3_C_2_) compared to the 900 W of laser beam power. This gives a more than 30% decrease in wear resistance. Taking into account the coatings produced with the laser beam powers of 900 W and 1200 W, these differences are much larger. In the least resistant coating (W-Cr), the weight loss was about 6 mg, while for the W-Cr/75% Cr_3_C_2_ coating, the weight loss was about 3 mg. Therefore, it can be concluded that doubling the laser beam power causes a deterioration of the wear resistance of the W-Cr coatings by about 75%, while in the W-Cr/75% Cr_3_C_2_ coating, the decrease in resistance is twice as low. The decrease in wear resistance caused by an increase in the laser beam power is associated with an increase in the proportion of iron in the surface layer produced. Increasing the amount of iron from the substrate reduces the percentage of the hard reinforcing phase in the coating responsible for high hardness and abrasion resistance. It can be concluded that an increase in abrasion resistance is related to the presence of carbide mesh, which was observed in the structure of coatings produced with a laser beam power of 600 W. 

**Figure 17:**
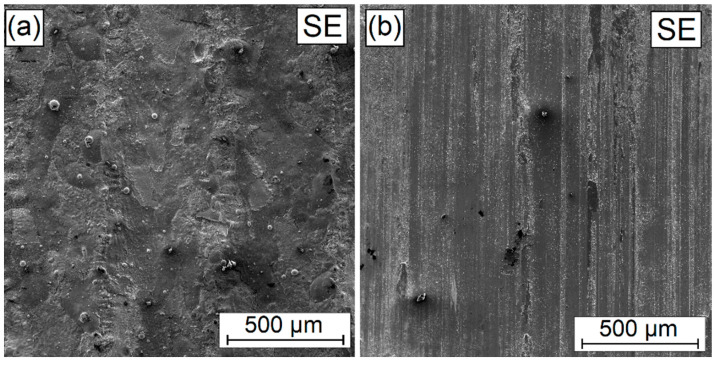
Surface condition before (**a**) and after (**b**) wear resistance test of W-Cr coating without reinforcing phase produced using laser processing of pre-coat with a laser beam power of 900 W.

**Figure 18:**
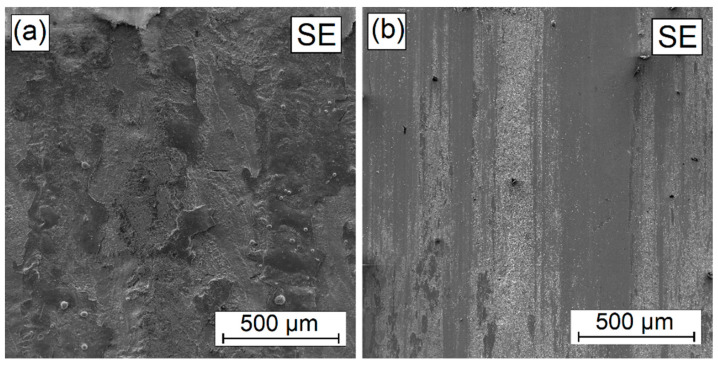
Surface condition before (**a**) and after (**b**) wear resistance test of W-Cr/25% Cr_3_C_2_ coating without reinforcing phase produced using laser processing of pre-coat with a laser beam power of 900 W.

**Figure 19:**
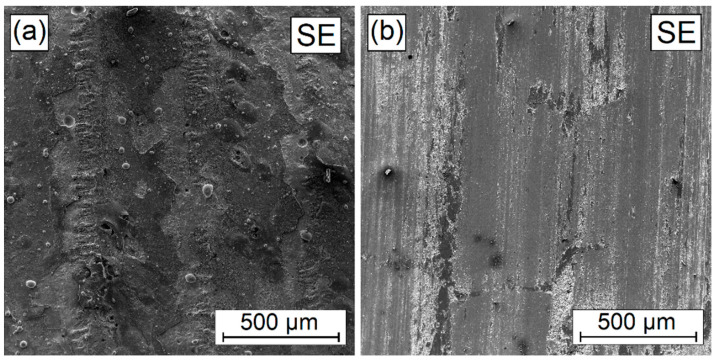
Surface condition before (**a**) and after (**b**) wear resistance test of W-Cr/50% Cr_3_C_2_ coating without reinforcing phase produced using laser processing of pre-coat with a laser beam power of 900 W.

**Figure 20:**
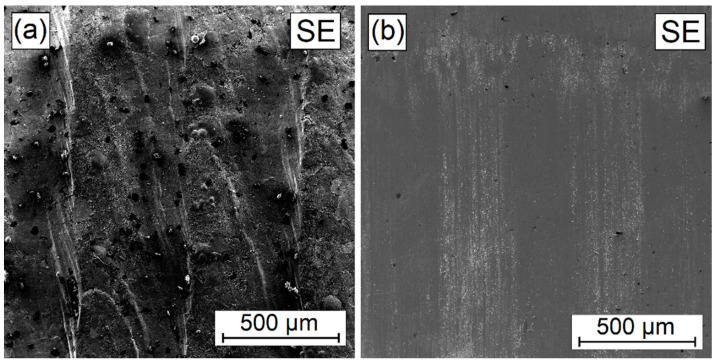
Surface condition before (**a**) and after (**b**) wear resistance test of W-Cr/75% Cr_3_C_2_ coating without reinforcing phase produced using laser processing of pre-coat with a laser beam power of 900 W.

**Figure 21:**
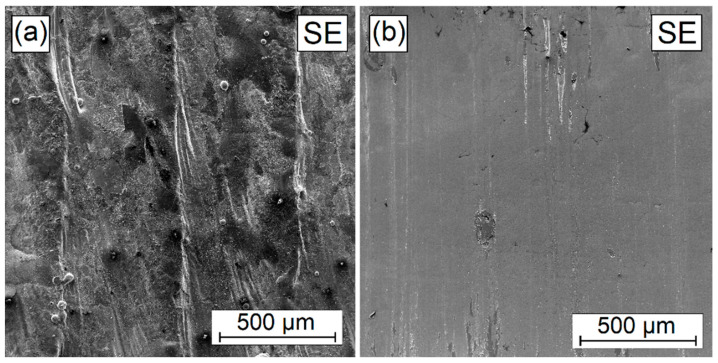
Surface condition before and after wear resistance test of 100% Cr_3_C_2_ coating without reinforcing phase produced using laser processing of pre-coat with a laser beam power of 900 W: (**a**) SE image of coating, (**b**) BSE image of wear trace.

In order to assess the condition of the surface and wear mechanism, tests were carried out under a scanning microscopy. The coatings characterized by intermediary properties, i.e., those produced using the power of the 900 W laser beam, were tested. The results are presented in Figure 17, Figure 18, Figure 19, Figure 20 and Figure 21. For comparison, the surface condition prior to the friction process is also presented. The fact that the initial surface condition was different for each sample might affect the results of the friction test; however, it should not have a significant impact on the assessment of wear resistance—and even more so if the coatings were created for use in friction conditions in the soil. As mentioned earlier, the coatings produced for agricultural tools do not require additional mechanical treatment such as grinding. Figure 17a shows the surface condition of the coating produced through laser processing of the W-Cr pre-coat. Figure 17b shows clear signs of wear, indicating low coating hardness. Grooving associated with the chipping of structural elements of higher hardness is also observed.

In the W-Cr coatings, into which 25% Cr_3_C_2_ (Figure 18) and 50% Cr_3_C_2_ (Figure 19) were introduced in addition to the mechanisms of microcutting and grooving, flaking (spalling) can also be observed. These symptoms intensify with an increase in the amount of the reinforcing phase. This is related to the difference in hardness of the carbide mesh and the matrix in it. Local differences in hardness result in the separation of harder areas from those characterized by lower hardness. 

On the other hand, flaking was neither observed among the W-Cr coatings containing 75% of the reinforcing phase (Figure 20) nor among the coatings produced by remelting chromium carbide alone with a steel substrate. Only the friction process was found to remove the unevenness resulting from the laser processing of the pre-coat. As for the W-Cr/75% Cr_3_C_2_ coating, no major surface defects were found; however, the wear surface of the 100% Cr_3_C_2_ coating was characterized by the occurrence of microcracks caused by the counter-sample pressure (Figure 21). Due to the unevenness on the coating, it is likely that there was a cyclical impact of frictional vapour, which caused the spread of microcracks. It was found to be a result of a friction effect because microscopic observations did not show the presence of so many microcracks in the coatings. 

### 3.4. Corrosion Resistance

**Figure 22:**
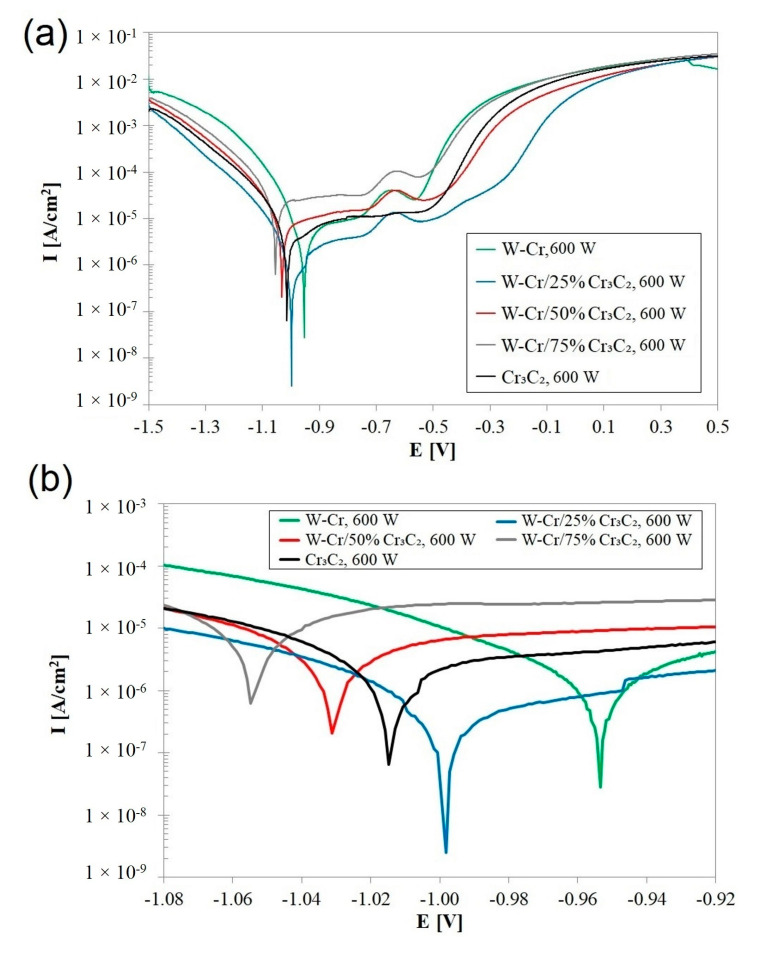
Corrosion resistance curves produced using 600 W: (**a**) full potentials range for each coatingd, (**b**) E_corr_ potentials range for each coating.

**Figure 23:**
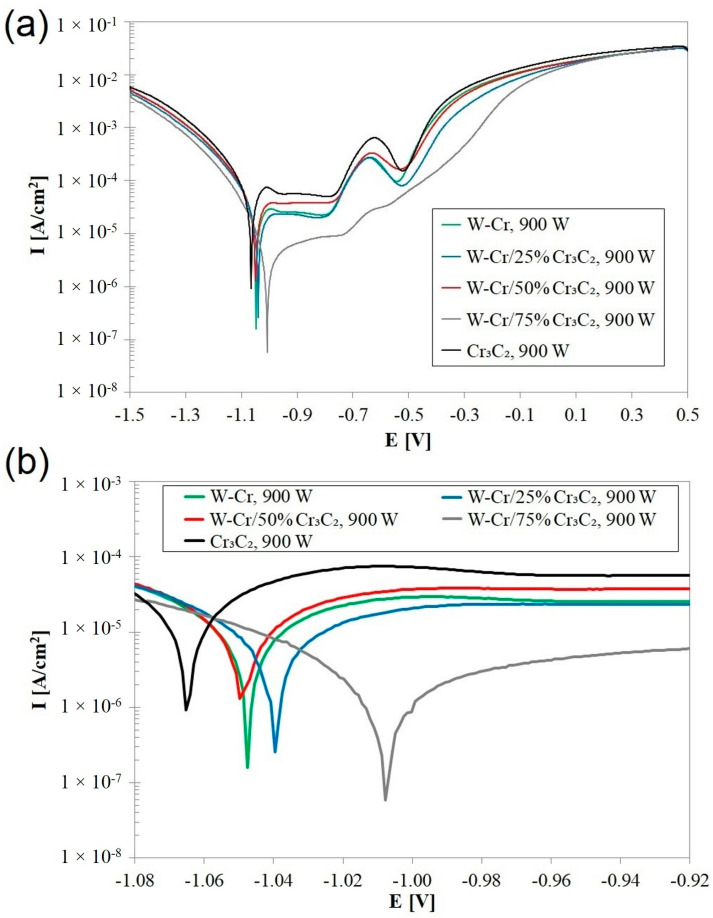
Corrosion resistance curves produced using 900 W: (**a**) full potentials range for each coatingd, (**b**) E_corr_ potentials range for each coating.

**Figure 24:**
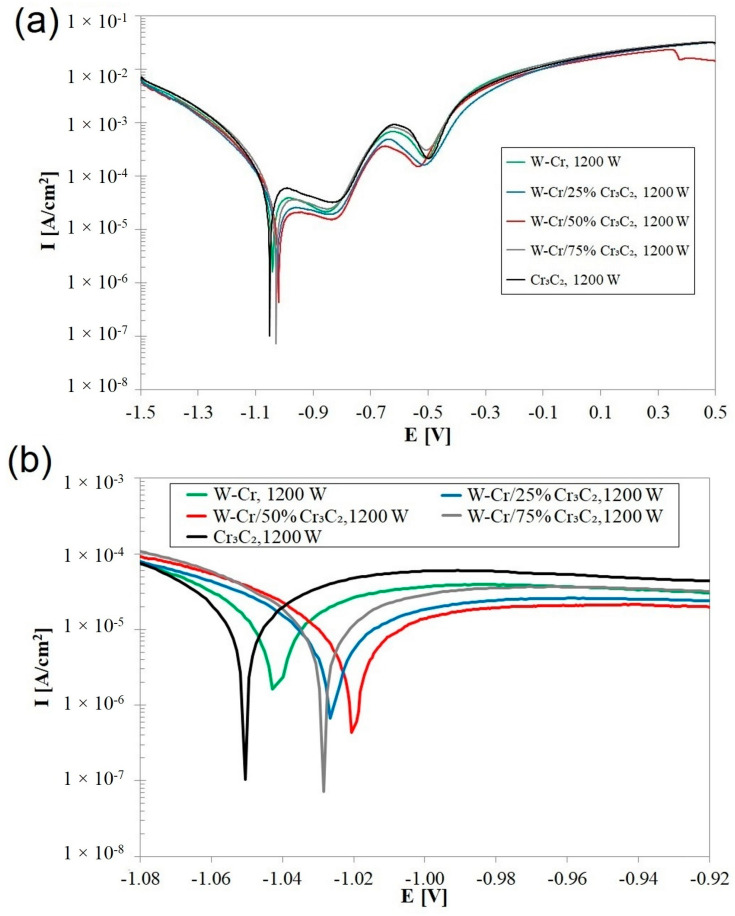
Corrosion resistance curves produced using 1200 W: (**a**) full potentials range for each coatingd, (**b**) E_corr_ potentials range for each coating.

Potentiodynamic corrosion tests were performed on the W-Cr, W-Cr/Cr_3_C_2_ and Cr_3_C_2_ coatings. The results of these tests in the form of potentiodynamic curves are presented in Figure 22, Figure 23 and Figure 24, while the numerical values determined through Tafel plot extrapolation are presented in Table 9, Table 10 and Table 11. The test results for individual surface layers were compared to the use of laser beam power. It was assumed that corrosion resistance is greater among plots that are shifted towards positive potential values. In order to better illustrate the shifts of individual plots, enlargements of the studied areas are attached. At the outset, however, it should be noted that the differences in corrosion resistance were not significant. Figure 22 shows corrosion curves obtained for the surface layers produced with a laser beam power of 600 W. The lowest laser beam power was found to cause the least mixing of the pre-coat with the substrate. This led to the highest corrosion resistance for the W-Cr coating. However, the use of the reinforcing phase alone, with a relatively low laser beam power, will result in a deterioration of corrosion resistance in comparison to the W-Cr coatings. With 600 W of laser beam power, it was found that using the W-Cr mixture reinforced with Cr_3_C_2_ particles in the amount of 50% and 75% is unfavorable. This may be related to an increase in the number of phases occurring and thus the formation of corrosion cells between materials with different electrochemical potentials. However, the 25% addition of Cr_3_C_2_ was proved to not significantly deteriorate corrosion resistance. The numerical values of corrosion current and corrosion potential of the coatings produced with a laser beam power of 600 W are presented in Table 9.

Figure 23 shows the corrosion curves obtained for surface layers produced at a laser beam power of 900 W. By analyzing the magnification of the corrosion potential area (Figure 23b), it can be concluded that the increase in power had a negative impact on the corrosion resistance of the W-Cr coating. This was due to the greater mixing of the pre-coat material with the steel substrate, which increased the proportion of iron, a metal that has a lower corrosion resistance than chromium and tungsten. In proportion to the corrosion resistance of the W-Cr coating, the corrosion resistance of the Cr_3_C_2_ coating also decreased, and its causes should be found in exactly the same mechanism. The corrosion resistance of the W-Cr/Cr_3_C_2_ coatings was between that of the pure W-Cr and the Cr_3_C_2_ coatings, but the differences in corrosion potential were too small to find any correlations. However, it should be emphasized that an increase in laser beam power during the production of these coatings led to a minimal deterioration of corrosion resistance in the coatings produced at a laser beam power of 600 W. However, it was found that a 75% addition of the reinforcing phase with a simultaneous increase in power to 900 W contributed to a minimal improvement in anti-corrosion properties. However, these changes are so small that no significant dependencies should be searched for. They may result from the quality of the pre-coat and are thus burdened with a certain error. The numerical values of the corrosion current and corrosion potential of the coatings produced with a laser beam power of 900 W are presented in Table 10.

Figure 24 shows the corrosion curves obtained for surface layers produced with a laser beam power of 1200 W. In this case, the results are very similar to those obtained with a laser beam power of 900 W. No significant changes were observed among the W-Cr and Cr_3_C_2_ coatings. However, it can be concluded that their resistance is lower than that of the W-Cr coatings reinforced with chromium carbides. As with the use of 900 W of laser beam power, the corrosion resistance was not found to depend significantly on the amount of the reinforcing phase. The modifications are rather negligible. The numerical values of the corrosion current and corrosion potential of coatings produced with a laser beam power of 600 W are presented in Table 11. 

**Figure 25:**
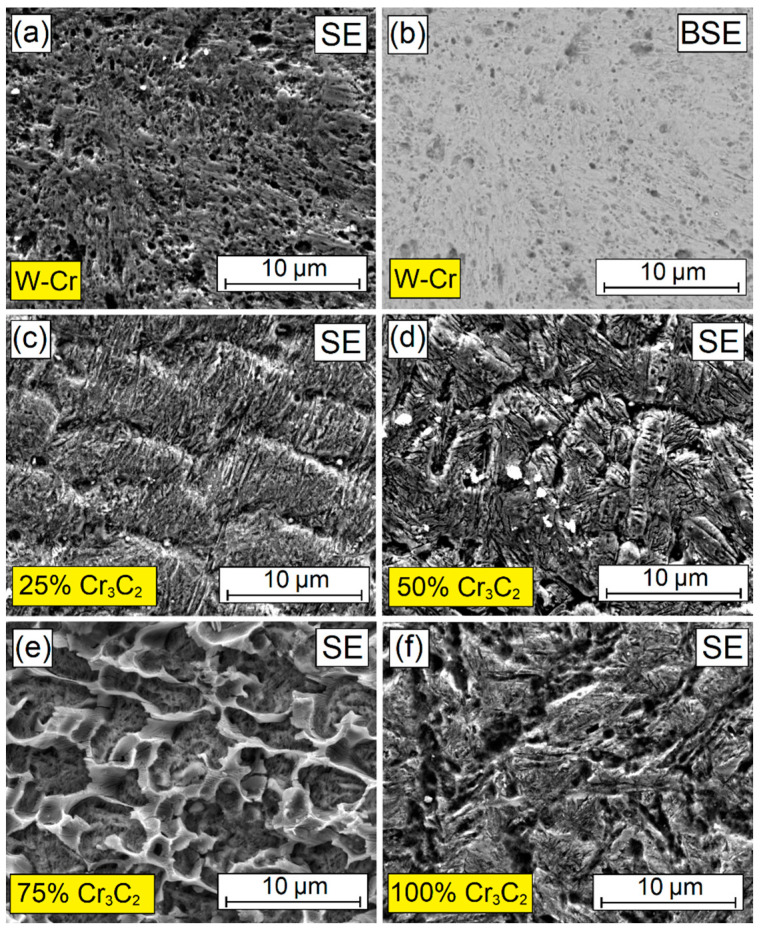
Surface after corrosion resistance of coatings produced using 900 W of laser beam power: (**a**) SE image of W-Cr coating, (**b**) BSE image of W-Cr coating, (**c**) SE image of W-Cr/25% Cr_3_C_2_ coating, (**d**) SE image of W-Cr/50% Cr_3_C_2_ coating, (**e**) SE image of W-Cr/75% Cr_3_C_2_ coating, (f) SE image of 100% Cr_3_C_2_ coating.

Similarly, as in wear resistance tests, observations of coating surfaces produced with a laser beam power of 900 W following corrosion tests were made. Macroscopic observations were carried out using a scanning electron microscope in SE contrast. Additionally, for the W-Cr coating, an image in BSE contrast is presented; the images, shown in Figure 25, are of the W-Cr coating (Figure 25a,b), W-Cr/25% Cr_3_C_2_ coating (Figure 25c), W-Cr/50% Cr_3_C_2_ coating (Figure 25d), W-Cr/75% Cr_3_C_2_ coating (Figure 25e) and 100% Cr_3_C_2_ coating (Figure 25f). Prior to the corrosion tests, the samples surfaces were prepared through grinding so that they were characterized by the same surface roughness. The corrosion resistance was found to be directly influenced by the microstructure obtained. With 600 W of laser beam power, the best corrosion resistance was demonstrated by the W-Cr coating. 

As can be seen in Figure 25a,b, the structure is homogeneous. The corrosive process resulted in the formation of small pits. However, due to the absence of various structural elements, this material does not undergo corrosion easily. An addition of 25% of the reinforcing phase to the W-Cr matrix caused slight changes (Figure 25c). Furthermore, there were no significant changes in the coating made by remelting the Cr_3_C_2_ pre-coat. In surface layers reinforced with 75% of Cr_3_C_2_ phase, obvious changes in the coating material caused by corrosion are visible. It is evident which structural elements are more corrosion resistant. Carbide mesh exhibits corrosion resistance, while numerous corrosion pits were observed in the matrix in it. It can therefore be concluded that a high content of the reinforcing phase, with all its advantages related to high hardness and wear resistance, is not resistant to a corrosive environment.

## 4. Conclusions

Based on the tests carried out for the W-Cr, W-Cr/Cr_3_C_2_ and Cr_3_C_2_ coatings, the following conclusions can be drawn:

1. It is possible to produce hard and wear-resistant coatings built from the W-Cr matrix and the Cr_3_C_2_ reinforcing phase, whereby the use of laser beam power in the range of 600–1200 W completely remelts the introduced reinforcing phase, forming a new structure. Thus, it is not possible to produce a composite coating, i.e., a coating in which the reinforcing phase is clearly separated from the matrix. This is an issue with great potential for further research, especially in the context of the use of larger reinforcing phase particles, a different type of laser or another laser processing method.
2. By carefully selecting laser beam parameters (mainly laser beam power) and the chemical composition of the pre-coat, it is possible to control the properties of the surface layers produced. These properties are largely dependent on the microstructure obtained.
3. Addition of the reinforcing phase in the form of chromium carbide particles significantly improves resistance to wear through friction and microhardness of the coatings produced. Increasing the amount of the reinforcing phase increases the mechanical properties; however, it may lead to a deterioration of the corrosion resistance of the coatings.
4. In the case of using the W-Cr coating without the reinforcing phase, an average microhardness of 620 HV0.1 was obtained for the laser beam power of 600 W. The addition of the Cr_3_C_2_ coating resulted in a gradual increase in microhardness from 750 HV0.1 (for 25% Cr_3_C_2_) to 880 HV0. 1 (for 50% Cr_3_C_2_), up to about 890 HV0.1 (for 75% Cr_3_C_2_). It can be concluded that the reinforcing phase has a positive effect on the microhardness.
5. The proposed technology to produce W-Cr/Cr_3_C_2_ coatings can be successfully used in mining and agriculture, where the tools operating in soil do not have to meet strict size criteria but are expected to work for as long as possible in difficult operating conditions.

## Figures and Tables

**Table 1:** Chemical composition of 145Cr6 steel used in the study [wt.%].

C	Mn	Si	P	S	Cr	Mo	Ni	V	Fe
1.35	0.61	0.32	0.025	0.023	1.45	0.15	0.20	0.20	bal.

**Table 2:** Parameters of laser processing.

No.	Type of Coating	Laser Power P [W]	Power Density q [kW/cm^2^]	Laser Beam Rate v [m/min]
1A	100% W-Cr	600	76.4	3.0
1B		900	114.6	3.0
1C		1200	152.9	3.0
2A	W-Cr/25% Cr_3_C_2_	600	76.4	3.0
2B		900	114.6	3.0
2C		1200	152.9	3.0
3A	W-Cr/50% Cr_3_C_2_	600	76.4	3.0
3B		900	114.6	3.0
3C		1200	152.9	3.0
4A	W-Cr/75% Cr_3_C_2_	600	76.4	3.0
4B		900	114.6	3.0
4C		1200	152.9	3.0
5A	100% Cr_3_C_2_	600	76.4	3.0
5B		900	114.6	3.0
5C		1200	152.9	3.0

**Table 3:** Average thicknesses of obtained coatings.

Pre-Coat/Laser Beam Power	600 W	900 W	1200 W
100%W-Cr	430 μm	580 μm	879 μm
W-Cr/25% Cr_3_C_2_	415 μm	620 μm	961 μm
W-Cr/50% Cr_3_C_2_	354 μm	697 μm	899 μm
W-Cr/75% Cr_3_C_2_	341 μm	682 μm	972 μm
100% Cr_3_C_2_	395 μm	587 μm	1006 μm

**Table 4:** Chemical composition (EDS) of the W-Cr coating. Designations according to Table 2.

Coating	No	Fe (wt.%)	Cr (wt.%)	W (wt.%)	C (wt.%)
1A	1	67.19	5.79	10.53	16.50
	2	71.53	4.90	4.60	18.97
	3	73.90	4.15	3.80	18.15
1B	1	79.19	3.65	5.09	12.07
	2	81.23	3.32	4.66	10.79
	3	82.56	3.24	4.13	10.08
1C	1	87.02	0.82	2.20	9.96
	2	86.16	0.81	2.52	10.51
	3	84.82	0.91	2.20	12.07

**Table 5:** Chemical composition (EDS) of the W-Cr/25% Cr_3_C_2_ coating. Designations according to Table 2.

Coating	No	Fe (wt.%)	Cr (wt.%)	W (wt.%)	C (wt.%)
2A	1	77.14	4.45	4.61	13.80
	2	75.69	4.82	4.85	14.63
	3	76.99	4.92	4.39	13.70
2B	1	76.89	3.22	3.72	16.17
	2	78.00	3.31	3.78	14.91
	3	78.54	3.54	3.72	14.21
2C	1	6748	2.06	3.17	27.29
	2	77.28	1.76	2.93	18.03
	3	74.26	1.45	2.71	21.58

**Table 6:** Chemical composition (EDS) of the W-Cr/50% Cr_3_C_2_ coating. Designations according to Table 2.

Coating	No	Fe (wt.%)	Cr (wt.%)	W (wt.%)	C (wt.%)
3A	1	64.92	15.66	7.30	12.12
	2	54.01	19.14	8.94	17.92
	3	66.14	12.01	6.06	15.79
3B	1	69.41	3.33	2.82	24.44
	2	77.14	3.64	3.09	16.14
	3	67.63	5.83	5.69	20.85
3C	1	74.68	1.91	2.52	20.88
	2	76.25	1.41	2.06	20.28
	3	76.04	1.20	2.25	20.51

**Table 7:** Chemical composition (EDS) of the W-Cr/75% Cr_3_C_2_ coating. Designations according to Table 2.

Coating	No	Fe (wt.%)	Cr (wt.%)	W (wt.%)	C (wt.%)
4A	1	62.34	15.51	4.19	17.96
	2	44.02	24.22	6.44	25.32
	3	60.31	14.96	3.85	20.87
4B	1	75.52	5.25	2.06	17.18
	2	78.10	5.53	2.28	14.09
	3	60.01	13.26	4.41	22.31
4C	1	65.14	7.80	3.62	23.44
	2	78.56	2.37	1.59	17.49
	3	74.30	5.03	2.62	18.04

**Table 8:** Chemical composition (EDS) of the Cr_3_C_2_ coating. Designations according to Table 2.

Coating	No	Fe (wt.%)	Cr (wt.%)	W (wt.%)	C (wt.%)
5A	1	55.93	17.91	0.16	26.00
	2	73.28	12.29	0.42	14.02
	3	53.69	17.97	0.14	28.21
5B	1	77.13	7.58	0.40	14.89
	2	80.71	6.10	0.39	12.80
	3	83.70	5.61	0.19	10.50
5C	1	78.14	4.47	0.79	16.60
	2	73.54	4.55	0.85	21.07
	3	71.88	4.96	0.46	22.70

**Table 9:** Corrosion resistance parameters of W-Cr/Cr_3_C_2_ coatings produced via laser processing using 600 W of laser beam power.

Coatings Number	Icorr [A/cm^2^]	Ecorr [V]
1A	1.13 × 10^−6^	−9.53 × 10^−1^
2A	1.01 × 10^−7^	−9.98 × 10^−1^
3A	2.02 × 10^−7^	−1.03 × 10^0^
4A	4.30 × 10^−7^	−1.05 × 10^0^
5A	9.35 × 10^−7^	−1.01 × 10^0^

**Table 10:** Corrosion resistance parameters of W-Cr/Cr_3_C_2_ coatings produced via laser processing using 900 W of laser beam power.

Coatings Number	Icorr [A/cm^2^]	Ecorr [V]
1B	3.11 × 10^−6^	−1.05 × 10^0^
2B	4.30 × 10^−6^	−1.04 × 10^0^
3B	7.15 × 10^−6^	−1.05 × 10^0^
4B	7.16 × 10^−7^	−1.01 × 10^0^
5B	1.03 × 10^−5^	−1.07 × 10^0^

**Table 11:** Corrosion resistance parameters of W-Cr/Cr_3_C_2_ coatings produced via laser processing using 1200 W of laser beam power.

Coatings Number	Icorr [A/cm^2^]	Ecorr [V]
1C	6.55 × 10^−6^	−1.04 × 10^0^
2C	2.94 × 10^−6^	−1.03 × 10^0^
3C	4.24 × 10^−6^	−1.02 × 10^0^
4C	1.64 × 10^−6^	−1.03 × 10^0^
5C	1.03 × 10^−5^	−1.05 × 10^0^

## Data Availability

Data available on request.

## References

[1] Burakowski T., Wierzchoń T. (2020). Surface Engineering of Metals. Principles, Equipment, Technologies.

[2] Ermakova A., Braithwaite J., Razavi J., Ganguly S., Berto F., Mehmanparast A. (2023). The influence of laser shock peening on corrosion-fatigue behaviour of wire arc additively manufactured components. Surf. Coat. Technol..

[3] Nathan S.R., Suganeswaran K., Kumar S., Thangavel P., Gobinath V.K. (2023). Investigations on microstructure, thermo-mechanical and tribological behavior of graphene oxide reinforced AA7075 surface composites developed via friction stir processing. J. Manuf. Process..

[4] Hou W., Ding Y., Huang G., Huda N., Shah L.H.A., Piao Z., Shen Y., Shen Z., Gerlich A. (2022). The role of pin eccentricity in friction stir welding of Al-Mg-Si alloy sheets: Microstructural evolution and mechanical properties. Int. J. Adv. Manuf. Technol..

[5] Cavaliere P. (2021). Laser Cladding of Metals.

[6] Aghili S.E., Shamanian M. (2019). Investigation of powder fed laser cladding of NiCr-chromium carbides single-tracks on titanium aluminide substrate. Opt. Laser Technol..

[7] Zhang D.-W., Lei T.C., Li F.-J. (2001). Laser cladding of stainless steel with Ni–Cr_3_C_2_ for improved wear performance. Wear.

[8] Wang H., Zhang S., Zhang C., Wu C., Zhang J., Abdullah A.O. (2018). Effects of V and Cr on Laser Cladded Fe-Based Coatings. Coatings.

[9] Wu Q., Li W., Zhong N., Gang W., Haishan W. (2013). Microstructure and wear behavior of laser cladding VC–Cr_7_C_3_ ceramic coating on steel substrate. Mater. Des..

[10] Bartkowski D., Młynarczak A., Piasecki A., Dudziak B., Gościański M., Bartkowska A. (2015). Microstructure, microhardness and corrosion resistance of Stellite-6 coatings reinforced with WC particles using laser cladding. Opt. Laser Technol..

[11] Bartkowski D., Bartkowska A., Jurči P. (2021). Laser cladding process of Fe/WC metal matrix composite coatings on low carbon steel using Yb: YAG disk laser. Opt. Laser Technol..

[12] Liang Y., Fu H., Xing Z., Guo X., Lin J. (2022). Effect of Cr_3_C_2_ Content on Microstructure and Properties of Laser Cladding Ti(C, B)/Ni Coatings. J. Mater. Eng. Perform..

[13] Jiang J., Hou W., Feng X., Shen Y. (2023). Oxidation resistant FeCoNiCrAl high entropy alloy/AlSi12 composite coatings with excellent adhesion on Ti-6Al-4 V alloy substrate via mechanical alloying and subsequent laser cladding. Surf. Coat. Technol..

[14] Shen Q., Xue J., Yu X., Zheng Z., Ou N. (2022). Triple-wire plasma arc cladding of Cr-Fe-Ni-Tix high-entropy alloy coatings. Surf. Coat. Technol..

[15] Pu J., Sun Y.-B., Wu L., He P., Long W.-M. (2022). Effect of CeO_2_ Content on Microstructure and Properties of Ni-Based Tungsten Carbide Layer by Plasma Arc Cladding. Coatings.

[16] Jonda E., Labisz K., Dobrzański L.A. (2016). Microstructure and properties of the hot work tool steel gradient surface layer obtained using laser alloying with tungsten carbide ceramic powder. Arch. Mater. Sci. Eng..

[17] Bartkowski D. (2021). Manufacturing Technology and Properties of Fe/TaC Metal Matrix Composite Coatings Produced on Medium Carbon Steel Using Laser Processing—Preliminary Study on the Single Laser Tracks. Materials.

[18] Młynarczak A., Jóźwiak K., Mesmacque G. (2003). Wear Resistance of Multiphase Diffusion Carbide Coatings. Adv. Eng. Mater..

[19] Młynarczak A. (2006). Early Stage of Diffusional Formation of Carbide Coatings on Steels. Adv. Eng. Mater..

[20] Borecki P., Młynarczak A. (2008). Modification of chromized diffusion carbide layer on tool steels using laser treatment. Arch. Metall. Mater..

[21] Wang S., Zhang S., Zhang C.H., Wu C.L., Chen J. (2018). Babar Shahzad, M. Effect of Cr_3_C_2_ content on 316L stainless steel fabricated by laser melting deposition. Vacuum.

[22] Wu H., Kong D. (2019). Effects of laser power on friction–wear performances of laser thermal sprayed Cr_3_C_2_–NiCr composite coatings at elevated temperatures. Opt. Laser Technol..

[23] Zong W., Zhang S., Zhang C., Wu C., Zhang J., Liu Y., Abdullah A.O. (2019). Preparation and Characterization of In Situ Carbide Particle Reinforced Fe-Based Gradient Materials by Laser Melt Deposition. Coatings.

[24] Han B., Li M., Wang Y. (2013). Microstructure and Wear Resistance of Laser Clad Fe-Cr_3_C_2_ Composite Coating on 35CrMo Steel. J. Mater. Eng. Perform..

[25] Maharajan S., Rex F.M.T., Ravindran D., Rajakarunakaran S. (2022). Erosive and corrosive wear performance and characterization studies of plasma-sprayedWC/Cr_3_C_2_ coating on SS316. Int. J. Appl. Ceram. Technol..

[26] Bartkowska A., Pertek A., Kulka M., Klimek L. (2015). Laser surface modification of boronickelized medium carbon steel. Opt. Laser Technol..

[27] (2013). Standard Korozja Metali i Stopów—Elektrochemiczne Metody Badań—Wytyczne Wykonania Potencjostatycznych i Potencjodynamicznych Pomiarów Polaryzacji (Corrosion of Metals and Alloys—Electrochemical Test Methods—Guidelines for the Performance of Potentiostatic and Potentiodynamic Polarization Measurements—Translation from Polish).

